# Ivonescimab monotherapy in an elderly patient with EGFR-TKI-resistant advanced lung adenocarcinoma and pericardial effusion: a case report and literature review

**DOI:** 10.3389/fimmu.2026.1728444

**Published:** 2026-05-12

**Authors:** Chen Zhu, Hui Yu, Ying Gu, Tingshu Jiang

**Affiliations:** Department of Respiratory and Critical Care Medicine, Yantai Yuhuangding Hospital, Yantai, Shandong, China

**Keywords:** advanced lung adenocarcinoma, EGFR-TKI resistance, elderly patient, ivonescimab, pericardial effusion

## Abstract

**Background:**

Ivonescimab is a first-in-class bispecific antibody targeting PD-1 and VEGF, showing promise in non-small cell lung cancer (NSCLC). This case report aims to explore the feasibility and efficacy of ivonescimab monotherapy in an elderly patient with EGFR-TKI-resistant advanced lung adenocarcinoma complicated by malignant pericardial effusion.

**Case presentation:**

A 77-year-old female with a 19-year history of postsurgical left lung adenocarcinoma was admitted with dyspnea and edema. Disease progression was observed after four cycles of chemotherapy (docetaxel plus nedaplatin) and subsequent EGFR-TKI therapy (icotinib and later furmonertinib combined with anlotinib). Upon readmission in February 2025 due to aggravated dyspnea, imaging revealed significant mediastinal tumor enlargement and a large pericardial effusion. Cytopathological examination of the pericardial fluid confirmed the presence of malignant cells, indicating TKI resistance. Given her advanced age and poor performance status precluding further chemotherapy, the patient was started on ivonescimab monotherapy (800 mg intravenously every 3 weeks). After six cycles of treatment, a follow-up CT scan demonstrated a marked reduction in the mediastinal tumor size and resolution of the pericardial effusion. Serum tumor markers, such as CYFRA 21–1 and proGRP, showed a decreasing trend. The treatment was well-tolerated with no significant adverse events observed during the follow-up periodstoryliter.

**Conclusion:**

This case suggests that ivonescimab monotherapy can achieve disease control with an acceptable safety profile in an elderly, heavily pretreated NSCLC patient with EGFR-TKI resistance and malignant pericardial effusion. It may represent a viable treatment option for this challenging population.

## Background

Lung cancer is the second most commonly diagnosed cancer and the leading cause of cancer-related mortality worldwide. According to the 2022 GLOBOCAN estimates, lung cancer accounts for approximately 1.8 million deaths annually (1). Non-small cell lung cancer (NSCLC) comprises over 80% of all lung cancer cases, and the majority (70%) are diagnosed at an advanced stage (stage III or IV) (2). In China, lung cancer remains a major public health burden, with 1.06 million new cases reported in 2022—accounting for 22.0% of all malignant tumors—and 733,300 deaths, representing 28.5% of all cancer-related fatalities (3). Ivonescimab is a first-in-class bispecific antibody that simultaneously targets programmed cell death protein 1 (PD-1) and vascular endothelial growth factor (VEGF) (4). Unlike conventional combination therapies that require separate administration of anti-PD-1/L1 and anti-VEGF agents, Ivonescimab exerts dual-targeting effects through synergistic mechanisms, enhancing therapeutic efficacy. Recently, our department treated a patient with EGFR-TKI-resistant advanced lung adenocarcinoma who achieved disease stabilization following ivonescimab monotherapy. We present a retrospective analysis of the patient’s clinical presentation, diagnosis, and treatment course.

## Case presentation

**Figure 1:**
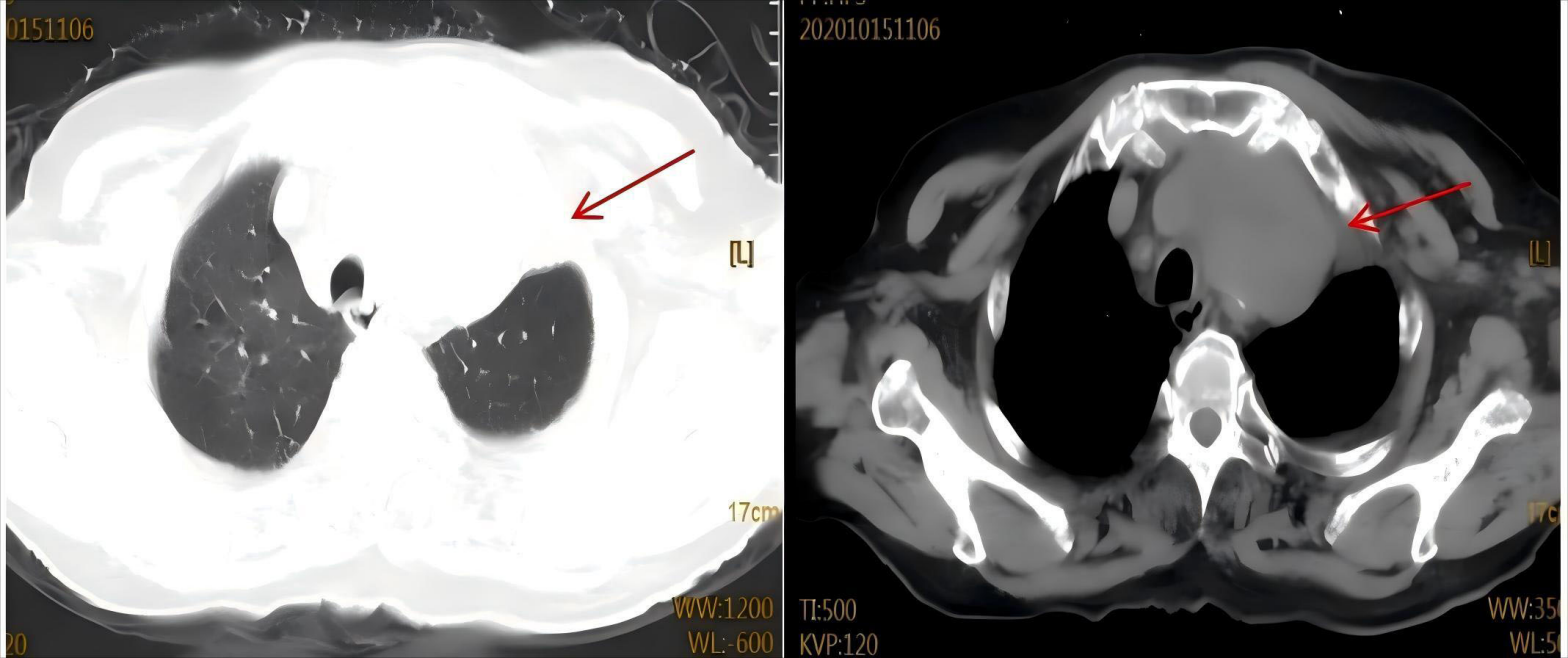
Baseline imaging at initial admission in october 2020.Chest CT scan reveals the anterior mediastinal mass (red arrow).

**Figure 2:**
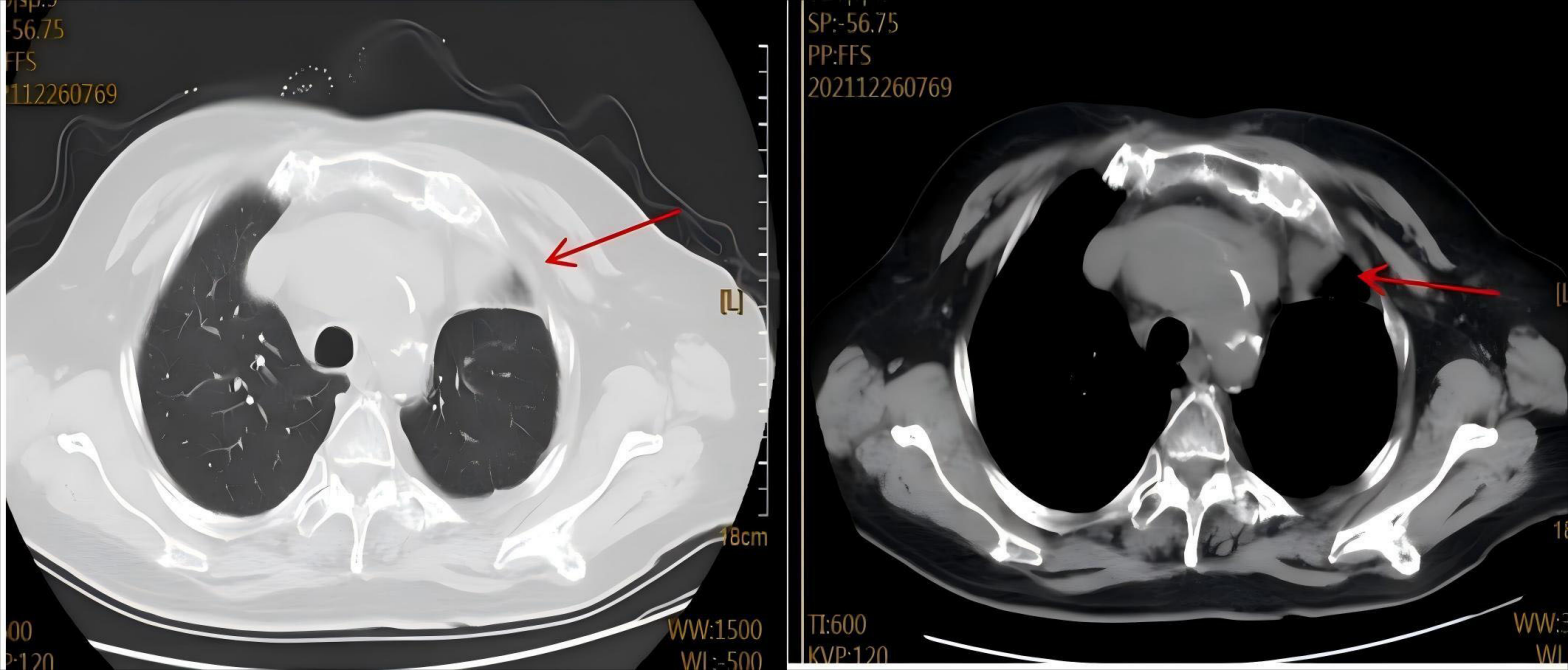
Treatment response assessment after first-line chemotherapy. Follow-up chest CT scan shows a significant reduction in the size of the anterior mediastinal mass (red arrow), consistent with a partial response.

**Figure 3:**
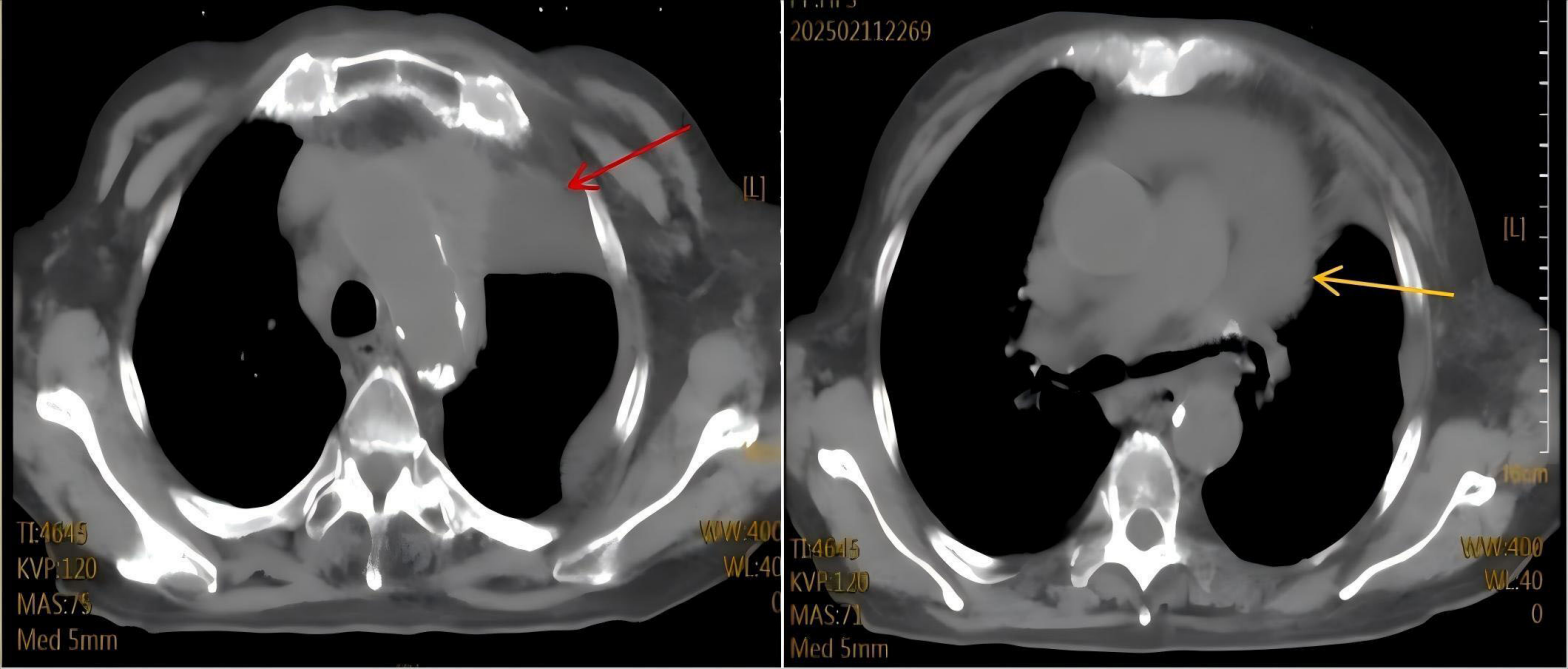
Disease progression upon readmission in february 2025.Chest CT scan demonstrates enlargement of the mediastinal tumor (red arrow), significant narrowing of the left main bronchus, and a newly developed large pericardial effusion (yellow arrow).

**Figure 4:**
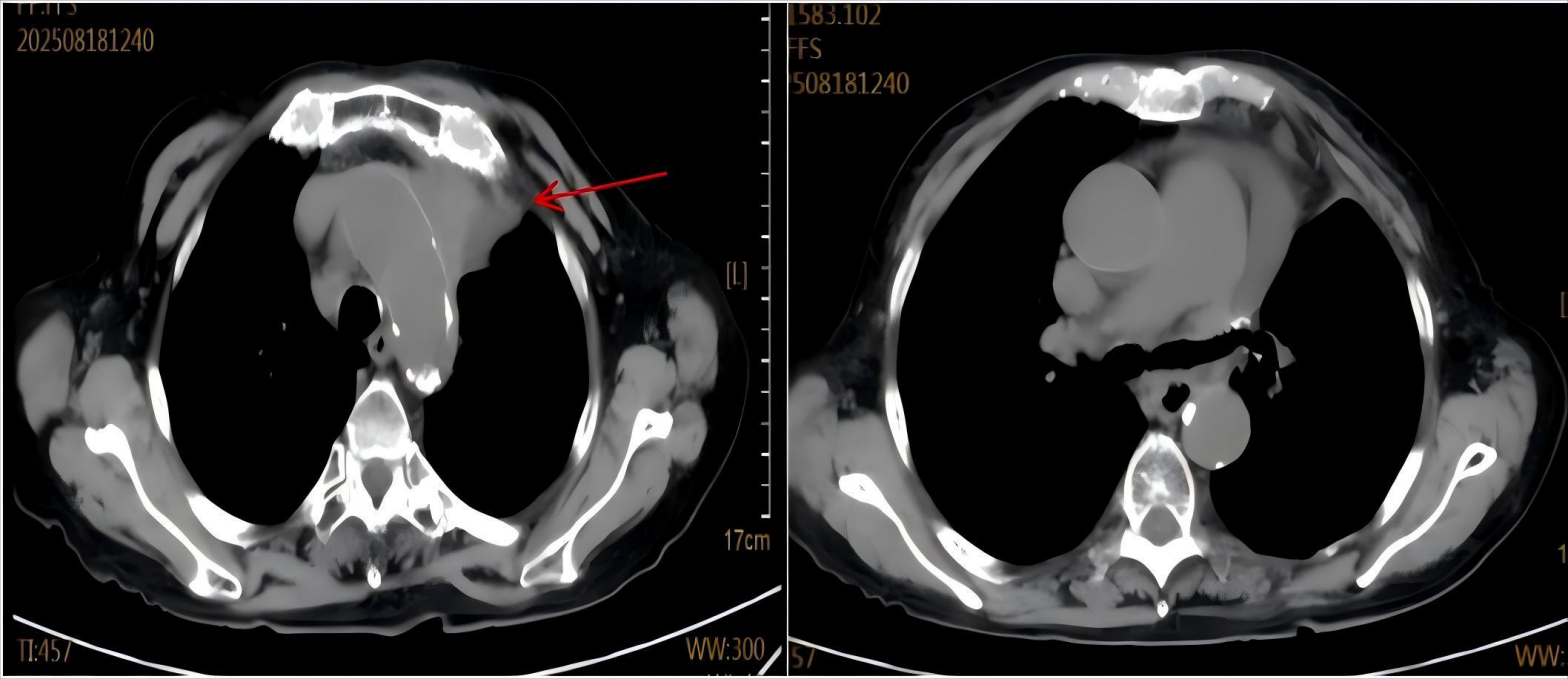
The August 2025 follow-up chest CT shows a marked reduction in the mediastinal tumor mass (red arrow) and resolution of the pericardial effusion.

**Figure 5:**
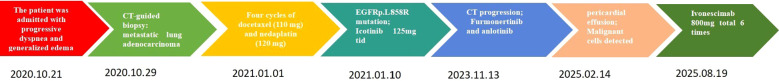
Schematic timeline of the patient’s clinical course and treatment history. The visual presentation of this timeline was informed by the case report format of Liu et al (16).

The patient was admitted on October 21, 2020, with a 10-day history of progressive dyspnea and edema involving the face, left upper limb, and bilateral lower extremities. Her symptoms began with exertional dyspnea, progressing to orthopnea and paroxysmal nocturnal dyspnea. Baseline characteristics are summarized in **Table 1**. The patient’s vital signs were as follows: temperature 36.8 °C, heart rate 108 beats/min, respiratory rate 23 breaths/min, and blood pressure 127/75 mmHg. The heart rhythm was regular, and no murmurs were heard. Pulmonary auscultation disclosed clear breath sounds bilaterally without crackles or wheezes. Abdominal examination was unremarkable. Initial chest CT revealed postoperative changes in the left lung and an anterior mediastinal mass (**Figure 1**). Cervical ultrasonography showed enlarged left cervical lymph nodes (max 2.4×1.0 cm). A subsequent CT-guided biopsy of the mediastinal mass confirmed adenocarcinoma, with immunohistochemistry positive for TTF-1 and CK7 and negative for CK5/6, consistent with metastatic lung adenocarcinoma. Staging workup, including contrast-enhanced CT/MRI and bone scan, showed no distant metastases. Although multidisciplinary consultation recommended genetic testing and chemoradiotherapy, the patient declined genetic testing and radiotherapy for financial reasons. From November 2020 to January 2021, the patient received four cycles of chemotherapy (docetaxel 110 mg + nedaplatin 120 mg,q3w). Follow-up CT showed a partial response (PR) of the mediastinal mass (**Figure 2**). She declined further intravenous chemotherapy but consented to genetic testing, which revealed an EGFR p.L858R mutation. Consequently, oral icotinib was initiated. In April 2021, a bone scan suggested left sacroiliac joint metastasis, leading to the addition of recombinant human endostatin. The disease remained stable until November 2023, when CT showed enlargement of left supraclavicular and mediastinal lymph nodes, indicating progression. Treatment was switched to furmonertinib plus anlotinib. In February 2025, the patient was readmitted with worsening dyspnea. Chest CT revealed an enlarged mediastinal mass, left main bronchus narrowing, and a large pericardial effusion (**Figure 3**). Pericardiocentesis yielded turbid yellow fluid with malignant cells confirmed by cytopathology. The patient’s symptoms improved significantly. Biochemical analysis showed LDH 1,779 U/L and CEA >1,000 ng/mL. Diagnostic challenges included difficulty obtaining tissue biopsy due to the patient’s age and poor performance status; however pericardial fluid cytology provided diagnostic material. Differential diagnosis considered infectious pericarditis and TKI-related cardiotoxicity, but the presence of malignant cells in pericardial fluid confirmed malignant pericardial effusion. Prognosis is generally poor for NSCLC with malignant pericardial effusion. NGS of the pericardial fluid revealed a high tumor mutational burden (TMB-H, 28.43 mut/Mb), microsatellite stability (MSS), and mutations in ATM (c.3747-1G>C) and TP53 (p.V157PfsTer23). The original EGFR p.L858R mutation was confirmed. PD-L1 testing was not performed due to financial constraints. Given her age, poor clinical condition, and ineligibility for further chemotherapy, the patient began ivonescimab monotherapy (800 mg, q3w) after thorough discussion with her family. She received six cycles from February to September 2025. A follow-up CT on August 19, 2025, showed tumor reduction, decreased bronchial compression, and near-resolution of the pericardial effusion, consistent with stable disease (**Figure 4**). Serum tumor markers (CYFRA 21-1, proGRP) showed a decreasing trend. No significant adverse events were observed. The clinical course is summarized in **Figure 5**.

**Table 1:** The patient’s baseline characteristics.

Characteristics	
Age	77
Gender	Female
Smoking history	No
Family history	No
Occupation	Farmer
Comorbidities	No
Genetic history	No
Previous medical history	left lung adenocarcinoma surgery 19 years prior

## Discussion

Ivonescimab, a first-in-class humanized tetravalent bispecific antibody, simultaneously targets PD-1 and VEGF-A. Its dual mechanism of action promotes anti-tumor immunity by inhibiting the PD-1 interaction while suppressing VEGF-driven angiogenesis, offering a novel therapeutic strategy for NSCLC (5). This coordinated approach may overcome the limitations of single-target therapies and address key resistance mechanisms within the tumor microenvironment. The landmark HARMONi phase III study demonstrated significant clinical benefit of ivonescimab plus chemotherapy in patients with EGFR-TKI-resistant NSCLC, with superior progression-free survival compared to chemotherapy alone (HR: 0.46; P < 0.001) (6). Earlier foundational work by Wang et al. in a Phase 1b study—the first human evaluation of ivonescimab monotherapy—confirmed its tolerable safety profile and revealed dose-dependent antitumor activity, particularly in PD-1-positive patients, with an ORR of 39.8% in immunotherapy-naïve advanced NSCLC (7). Furthermore, Zhao et al.’s Phase II trial showed the efficacy and safety of ivonescimab combined with chemotherapy in metastatic NSCLC, underscoring its broad applicability across treatment lines (4) However, the role of immunotherapy in EGFR-mutant NSCLC remains controversial. Unlike EGFR-wild-type tumors, EGFR-mutant NSCLC typically exhibits low tumor mutational burden (TMB), low PD-L1 expression, and an immunosuppressive tumor microenvironment, which are associated with poor response to immune checkpoint inhibitors (ICIs).Dual PD-1/VEGF blockade may overcome resistance in this patients. In the present case, as shown in **Figures 1**, **4**, the patient’s achieved a partial response after initial chemotherapy (**Figure 2**) but subsequently progressed following sequential treatment with second- and third-generation EGFR-TKIs. Upon readmission, imaging (**Figure 3**) demonstrated enlargement of the mediastinal tumor and a newly developed large pericardial effusion. The development of malignant pericardial effusion confirmed acquired resistance to EGFR-TKIs. Given her advanced age, poor performance status, and ineligibility for further chemotherapy, ivonescimab monotherapy was selected based on its favorable safety profile and dual mechanism of action. Prior to initiating immunotherapy, the patient’s large pericardial effusion was managed according to established guidelines for thoracic drainage (8), which provided safe and effective symptom relief. After six cycles of ivonescimab, follow-up imaging (**Figure 4**) revealed significant tumor reduction and near-complete resolution of the pericardial effusion, indicating disease stabilization. Malignant pericardial effusion in NSCLC is a poor prognostic sign. Complete resolution after ivonescimab monotherapy, without repeated drainage, is noteworthy and suggests effective control of serous effusions. Notably, this clinical improvement occurred without significant adverse events, further supporting the manageable safety profile of the drug. Third-generation EGFR-TKIs remain the standard of care for EGFR-mutant NSCLC; however, acquired resistance is inevitable (9, 10). The underlying mechanisms are complex and extend beyond secondary genetic mutations. Emerging evidence suggests that protein post-translational modifications (PTMs), such as phosphorylation and ubiquitination, regulate immune checkpoint molecules and modulate immunotherapy response (11). Additionally, caspase family proteases exhibit non-cell death functions, including immune homeostasis regulation and metabolic reprogramming, which may impact tumor progression and treatment response (12). Collectively, these findings highlight the multifaceted nature of resistance mechanisms within the tumor microenvironment.

Beyond bispecific antibodies, other immunotherapeutic strategies are advancing in NSCLC. Toripalimab, the first domestically developed anti-PD-1 antibody in China, has demonstrated efficacy in NSCLC (13). These developments continue to expand the therapeutic landscape for NSCLC patients. Following progression on first-line third-generation EGFR-TKIs, treatment options include platinum-based chemotherapy combined with bevacizumab or with sintilimab and bevacizumab (14), as well as ivonescimab combined with pemetrexed and carboplatin (6). After failure of both EGFR-TKI and platinum-based chemotherapy, amivantamab represents an alternative option, having received approval for this specific patient population (15).

This case provides clinical evidence supporting ivonescimab monotherapy as a viable treatment strategy, particularly for elderly patients with poor performance status who are ineligible for standard chemotherapy. The simultaneous blockade of immune checkpoints and angiogenesis pathways offers a compelling rationale in the setting of EGFR-TKI resistance. This study has limitations inherent to a single-case report, including limited generalizability and a relatively short follow-up period, which precludes comprehensive assessment of long-term outcomes. Larger prospective studies are warranted to validate the role of ivonescimab monotherapy in this challenging patient population. whilefavormetrecheckcheck.

## Data Availability

The original contributions presented in the study are included in the article/supplementary material. Further inquiries can be directed to the corresponding author.
